# Incidence of gynaecological (pre-)malignancies and endometrial activity in transmasculine and gender diverse individuals using testosterone: a retrospective, single-centre cohort study

**DOI:** 10.1016/j.eclinm.2025.103248

**Published:** 2025-05-12

**Authors:** Asra Vestering, Wouter L.J. van Vugt, Alison M. Berner, Malou L.H. Snijders, Martin den Heijer, Freek A. Groenman, Judith A.F. Huirne, Chantal M. Wiepjes, Norah M. van Mello

**Affiliations:** a Department of Obstetrics and Gynaecology, Amsterdam University Medical Center, Location Vrije Universiteit Amsterdam, the Netherlands; b Amsterdam Reproduction and Development Research Institute, Amsterdam, the Netherlands; c Center of Expertise on Gender Dysphoria, Amsterdam University Medical Center, the Netherlands; d Barts Cancer Institute, Queen Mary University of London, United Kingdom; e Gender Identity Clinic, Tavistock and Portman NHS Foundation Trust, London, United Kingdom; f Department of Pathology, Amsterdam University Medical Center, Amsterdam, the Netherlands; g Department of Endocrinology, Amsterdam University Medical Center, Location Vrije Universiteit Amsterdam, the Netherlands

**Keywords:** Transgender health, Testosterone therapy, Uterus, Ovaries, Gynaecological cancer, Endometrial activity

## Abstract

**Background:**

The number of transmasculine and gender diverse (TMGD) individuals that choose to postpone or refrain from surgical intervention to remove their internal gynaecological organs has been increasing. However, the safety of exogenous testosterone use in the presence of the reproductive organs, i.e. the risk of gynaecological malignancies remains unclear. This study aims to evaluate the incidence of gynaecological (pre-)malignancies in a nationwide cohort of TMGD individuals using testosterone treatment.

**Methods:**

This retrospective cohort study conducted at the Amsterdam University Medical Centre in the Netherlands, included transmasculine and gender diverse (TMGD) individuals receiving testosterone at our clinic between February 17, 1972 and December 3, 2018. Data from medical records were linked to the national pathology database to acquire diagnoses related to gynaecological cancer or gynaecological pathologies with malignant potential. TMGD individuals assigned female at birth who received testosterone were included, excluding those last seen before 1991. Based on observed and expected cases, age-adjusted standardised incidence ratios (SIR) were calculated to assess relative risk compared to the general population assigned female at birth.

**Findings:**

The cohort comprised 1955 TMGD individuals. Median age at start of gender-affirming hormone therapy was 21 years (interquartile range [IQR] 18–29). Prior to testosterone treatment 21·1% (413/1955) had used puberty suppression. Median duration of testosterone usage was 1·7 years (IQR 1·4–2·4) before hysterectomy and oophorectomy and 3·1 (2·3–5·4) before vaginal and/or vulvar surgery or biopsy. Median age at time of surgery or biopsy was 24 years (IQR 20–33) for uterine and ovarian histopathology acquisition and 29 (IQR 22–39) for vaginal and vulvar histopathology acquisition. No gynaecological malignancies were found, precluding SIR calculation. Expected incidence was 0·26 or less for all cancer types. One ovarian borderline tumour, one case of simple endometrial hyperplasia and one case of vulvar intraepithelial neoplasia III (VIN3) were detected. Based on the expected number of >VIN2 cases in our cohort (4·4) the age-adjusted standardised incidence ratio for > VIN2 was 0·23 (95% CI: 0·01–1·12).

**Interpretation:**

This is the largest cohort to date reporting on gynaecological histopathologic findings in TMGD individuals using testosterone. Based on these findings we can conclude that the risk of gynaecological malignancies is not increased in TMGD individuals using testosterone for a relatively short period of time compared to the general population assigned female at birth. However, to determine the long-term effects of testosterone on gynaecological organs, and counsel patients appropriately, studies with longer follow-up of individuals retaining these organs are needed.

**Funding:**

None.

**Research in context**

**Evidence before this study**

We conducted a comprehensive search of the PubMed database for literature published up to March 28, 2025. The search terms included *transmasculine individuals*, *gender dysphoria*, *masculinising hormone therapy*, *testosterone*, *androgen*, *reproductive organs*, *gynaecological malignancy*, and *histopathology*, along with their synonyms. Studies were included if they focused on the risk of gynaecological malignancies or pathology with malignant potential in transmasculine and gender diverse (TMGD) individuals using testosterone as gender-affirming hormone therapy (GAHT), without language restrictions. We also reviewed references cited in identified studies. This search specifically excluded cervical malignancy and cervical intraepithelial neoplasia, as these topics were addressed in a separate study due to their extensive scope. Our search returned 57 results, predominantly comprising case reports of endometrioid adenocarcinoma, atypical endometrial hyperplasia, ovarian cancer, and vaginal or vulvar carcinoma. Limited literature was found regarding certain pathologies with malignant potential, such as vulvar lichen sclerosus. Overall, these studies reported only isolated cases of gynaecological malignancies in TMGD individuals using testosterone. No studies provided comparative data enabling risk assessment of these malignancies relative to the general population assigned female at birth.

**Added value of this study**

As the population of TMGD individuals using testosterone who retain their reproductive organs grows, understanding gynaecological malignancy and pathology risks becomes increasingly important. To our knowledge, this study represents the first large-scale investigation of gynaecological malignancy risk in this population, with a cohort of 1955 TMGD individuals exposed to testosterone over several years. No cases of endometrial, ovarian, vaginal, or vulvar cancer were observed. Furthermore, the age-adjusted incidence of high-grade vulvar intraepithelial neoplasia in this cohort was lower than expected based on national incidence rates in the general population assigned female at birth.

**Implications of all the available evidence**

These findings suggest that short-term testosterone use among relatively young TMGD individuals is not associated with an increased risk of gynaecological malignancies compared to the general population assigned female at birth. However, the relatively young median age of this cohort must be considered. Future research should include prospective studies on cancer risk and screening in TMGD individuals who retain their reproductive organs for extended periods of time, incorporating factors such as hereditary cancer predisposition and HPV status for a more nuanced risk assessment.

## Introduction

Transmasculine and gender diverse (TMGD) individuals identify as male, non-binary or otherwise gender-nonconforming and were assigned female at birth (also referred to as registered female at birth).^1^ Gender-affirming hormone therapy (GAHT) with exogenous testosterone is used to induce masculinisation and align physical characteristics with their gender identity and/or gender expression.^2^ Despite widespread usage of testosterone in TMGD individuals, data regarding the long-term histological effects on the reproductive organs are scarce.^3^

In addition to GAHT, medical transition may involve gender-affirming surgeries (GAS). In recent years, increased acceptance of the transgender community has led to less restrictive laws in many countries.^4^ Until 2014, removal of reproductive organs was mandated as part of a change of legal gender in the Netherlands. Subsequently, a growing number of TMGD individuals opt to retain their reproductive organs or postpone GAS, resulting in a decrease in hysterectomy rates at the largest gender identity clinic in the Netherlands from 80% to 42%.^5^ Coupled with a reported global rise in the visibility and disclosure of transgender, non-binary, and gender diverse identities –particularly among younger cohorts– this has led to an increasing number of individuals initiating GAHT, resulting in more frequent and prolonged exposure of gynaecological organs to testosterone.^2^

Testosterone therapy, while generally considered safe, has raised concerns among some about the potential risks of endometrial hyperplasia and malignancy.^6^ Testosterone may aromatise to oestradiol, or directly affect the endometrium via androgen receptors, potentially inducing proliferation.^7^ However, in vitro studies suggest that androgens suppress endometrial activity, reducing risk of abnormal proliferation, and a cohort study found significantly reduced endometrial thickness in TMGD individuals exposed to testosterone.^8^^,^^9^ Studies on endometrial histology in TMGD individuals present conflicting results, with reports of active endometrium varying widely.^10^, ^11^, ^12^ Reassuringly, only isolated cases of endometrioid adenocarcinoma and atypical endometrial hyperplasia have been documented,^13^^,^^14^ and US cancer database studies have not shown grossly elevated rates, though these could not confirm sex assigned at birth or accurately code for trans individuals.^15^^,^^16^

Regarding ovarian tissue, several studies found morphological changes reminiscent of those found in polycystic ovaries.^6^^,^^12^^,^^17^ Instances of ovarian cancer in TMGD individuals using testosterone have been discussed in limited case reports.^18^, ^19^, ^20^, ^21^

Two case reports documented a case of vaginal carcinoma in one TMGD individual and vulvar carcinoma in another.^22^^,^^23^ No additional literature on vulvar pathology, such as lichen sclerosus, in TMGD individuals was found.

Thus, the impact of testosterone usage on gynaecologic tissue in TMGD individuals and its association with a risk of (pre-)malignant pathology remains unclear. While literature exists, prior studies are often small-scale, contradictory, and limited in number. Therefore, in this study we aimed to characterise histopathology from gynaecological organs in a large retrospective cohort of TMGD individuals using testosterone treatment. We compared the incidence of gynaecologic malignancies and conditions with malignant potential (i.e. endometrial atypia, borderline ovarium tumour, vaginal/vulvar intraepithelial neoplasms, lichen sclerosus) in TMGD individuals with the general population assigned female at birth. This is excluding cervical cancer and its precursors, as these were addressed in a separate study due to the extensive scope of this topic.^24^ Additionally, we investigated specific factors associated with active endometrium, to increase our understanding of the histopathological changes and potential risk of endometroid carcinoma in this unique cohort.

## Methods

### Study design & population

In this retrospective cohort study we identified all individuals who visited the gender identity clinic of Amsterdam UMC between February 17th 1972 and December 31st 2018.

This cohort has been previously described as the Amsterdam Cohort of Gender Dysphoria (ACOG).^2^ In the Netherlands, more than 95% of all transgender individuals seeking gender-affirming care visited our centre for psychological, endocrine or surgical treatment at that time.^2^ Inclusion criteria for the current study were: being assigned the female sex at birth and having a minimum duration of testosterone usage of one year (to ensure adequate dosing of hormonal treatment). Individuals who used systemic oestrogens as part of GAHT or hormonal replacement therapy during the duration of this study were excluded, with the exception of those using oestrogen-containing pills as contraceptive measure. This database was then linked to the Nationwide Network and Registry of Histopathology and Cytopathology in the Netherlands (PALGA), a national database containing all pathology reports since 1991.^25^ Therefore, individuals who had their last visit at our clinic before 1991 were excluded to prevent missing any (pre)-malignancies diagnosed before 1991.

TMGD individuals generally received testosterone treatment as a standalone hormonal treatment. For individuals who were younger than 18 years at start of hormone treatment, testosterone was often preceded by a gonadotropin-releasing hormone agonist (GnRHa). Various preparations of testosterone were prescribed: intramuscular injections of testosterone esters or undecanoate (Sustanon® and Nebido®); transdermal testosterone gel (AndroGel® and Tostran®); or undecanoate capsules (Andriol®). Some individuals also received progesterone treatment, or a gonadotropin-releasing hormone agonist for prevention of uterine bleeding or other hormonal contraceptives.

### Ethical considerations

This study adheres to the EQUATOR Network's STROBE guidelines for reporting observational studies.^26^ The study protocol was reviewed by the ethical review board of the VU University Medical Center Amsterdam, who concluded that the Medical Research Involving Human Subjects ACT (WMO) did not apply to this study. Necessity for informed consent was waived considering the retrospective design and large study population. All data were processed anonymously.

### Data collection

Medical data were retrieved from health records, including medical history, age at start of hormone treatment, any type of hormonal treatment, endocrine laboratory results and information on gender-affirming surgeries. Data were, if available, retrieved from records of visits at start of testosterone treatment, and thereafter at two, five and every ten years of follow-up. Data regarding gynaecological histopathology of uterine, ovarian, vaginal and vulvar tissue (i.e. type of histopathology, histopathological diagnosis and date of diagnosis) were obtained from the PALGA database. Data regarding cervical cancer and cervical intraepithelial neoplasia were also obtained but have been addressed in a separate study due to the extensive scope of this topic.^24^

### Statistical analysis

STATA Statistical Software, version 17 (Statacorp, College Station, TX, USA) and OpenEpi version 3·01 (www.OpenEpi.com) were used for statistical analyses.

Cohort characteristics are presented as mean ± standard deviation when normally distributed, median (interquartile range or range) when not normally distributed, or as frequencies and percentages where appropriate. Distribution was assessed using histograms. Only data prior to the (latest) date of surgery or biopsy (e.g. usage of other hormone treatment) were included in the analysis. Mean testosterone and oestradiol levels were calculated by averaging all measurements during GAHT, excluding the measurement at start GAHT. Mean BMI during treatment was calculated similarly as hormonal levels. Duration of testosterone usage until time of pathology was calculated for each organ separately, from the first known date of start of testosterone until date of histopathological specimen retrieval (biopsy or surgery). Similarly, follow-up time, which equals testosterone exposure time, was defined as years from start of testosterone treatment until the organ-specific study endpoint (i.e. until hysterectomy, oophorectomy, colpectomy or vulvar biopsy for uterine, ovarian, vaginal or vulvar pathology, respectively), date of death or end of the study period (December 31, 2018). In individuals who had started GAHT prior to their first visit at our clinic, the first known start date of GAHT was used to calculate treatment duration.

Total cohort follow-up time per organ (person years) was calculated by summing the follow-up time until the organ-specific study endpoint per age category, for all individuals. This was used to calculate age-adjusted standardised incidence ratio (SIR), using the observed and expected cases of ovarian, endometrial, vaginal and vulvar (pre)malignancies in our cohort. The number of expected cases of malignancies in our cohort were based on age-specific incidence rates for the general population assigned female at birth from the Netherlands Comprehensive Cancer Organisation (IKNL). Since the IKNL also generates gynaecological malignancy incidence rates using data from the same source (PALGA), this allowed for a reliable comparison between observed and expected cases.

To calculate 95% confidence intervals, the normal approximation of the Poisson distribution was applied. Negative values were truncated to align with the data's inherent characteristics. Age-specific incidence rates for vulvar and vaginal premalignancies (intra-epithelial neoplasia) were calculated using data obtained from PALGA public database and Statistics Netherlands.^27^ Subsequently, the expected cases of premalignancies in our cohort could be calculated and compared to the observed cases. SIRs with 95% confidence interval (95% CI) were calculated using a mid-exact P test.

To further investigate potential factors associated with endometrial activity, multiple logistic regression analysis was conducted, including BMI, type of testosterone, age at time of surgery or biopsy, use of puberty blockers prior to testosterone treatment and ovarian activity. Endometrial activity was defined as any mention of proliferative or secretory endometrium, as well as any other unspecified references to activity. In contrast, inactivity was recorded when specifically mentioned in the textual pathology report as inactivity or atrophy. Ovarian activity was defined as showing signs of recent ovulation, including the presence of a corpus luteum, corpus albicans, graafian follicle or follicles in various developmental stages. Before 2017, it was advised to discontinue testosterone treatment for two to six weeks prior to surgery to reduce the risk of thrombosis, potentially leading to recurrence of the menstrual cycle and thus induce endometrial proliferation. Therefore, the date of pathology (before or after 2017) was included as additional potential confounder in the model. Since only documented use of other hormonal treatments (i.e. progesterone, combined oestrogen/progesterone or GnRH-agonists) was known and the absence of combined treatment could not be confirmed, individuals with known other treatment were excluded. To minimise categories within independent variables, only Testosterone injections and gel were included, tablets were excluded as they are no longer prescribed.

### Role of the funding source

There was no funding source for this study. AV, WLJvV, CMW and NMvM had full access to the data. NMvM had final responsibility for the decision to submit for publication.

## Results

**Fig. 1:**
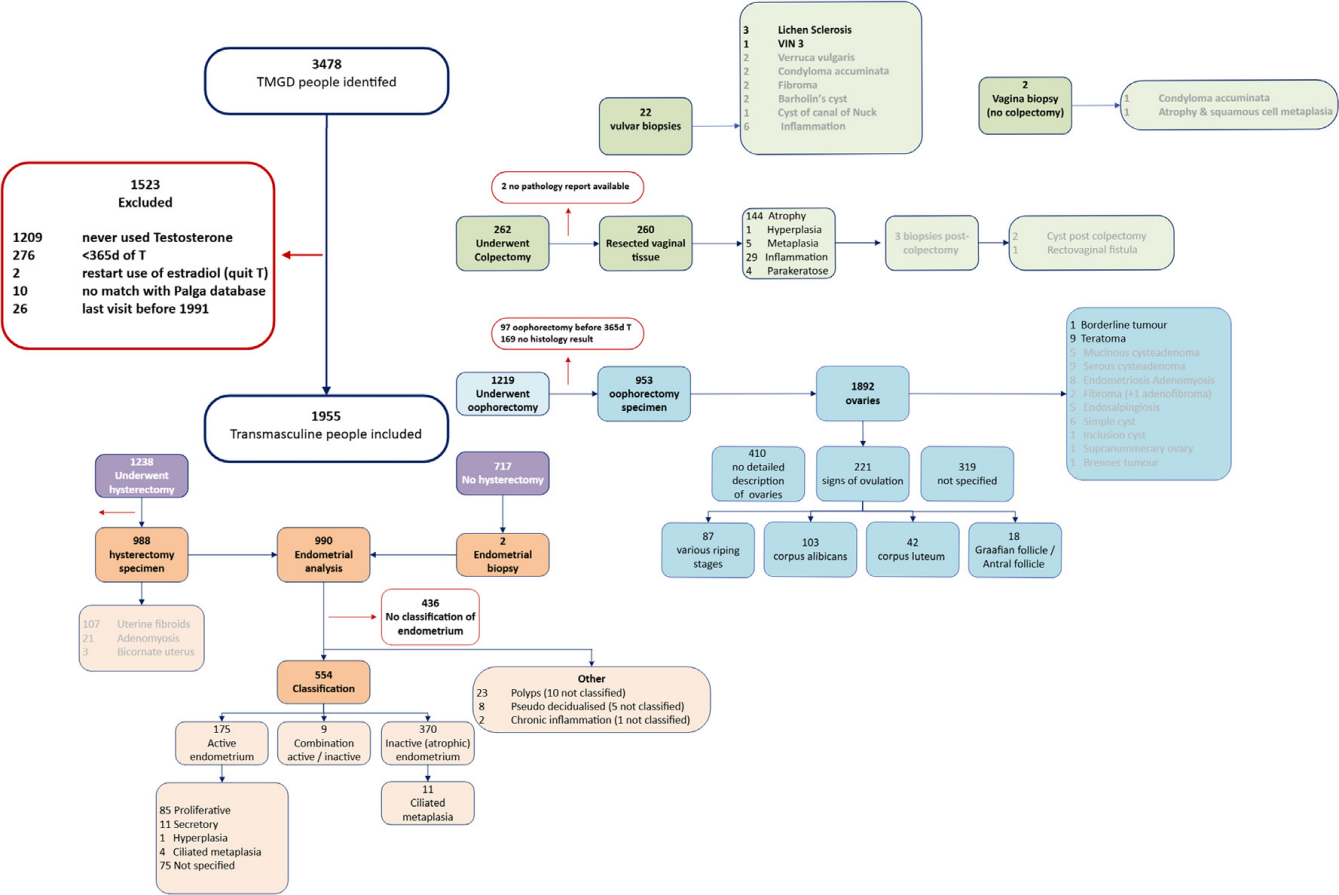
Study flowchart. TMGD, transmasculine and gender diverse. T, testosterone. VIN, vulvar intraepithelial neoplasia.

The cohort comprised a total of 3478 TMGD individuals. After exclusion, 1955 individuals were included in the current study (Fig. 1). Cohort characteristics are displayed in Table 1. Median age at start of gender-affirming hormone therapy was 21 years (interquartile range [IQR] 18–29). Prior to testosterone treatment 21·1% (413/1955) had used puberty suppression for a median duration of 9 months (IQR 5·7–24 months). For those who did not use puberty suppression, median serum testosterone and oestradiol levels were within the normal female range at initiation of GAHT. During GAHT median testosterone concentrations (21 nmol/L (IQR 13–32)) were within the therapeutic range. Median duration of testosterone usage for the overall cohort was 5·1 years (IQR 2·4–14·5).

**Table 1:** Study cohort characteristics.

	Sample size (N, total)^a^	Median (IQR) unless stated otherwise	
**Age at start testosterone, years**	1955	21	(18–29)
		Range 14–62	
**Puberty suppression before start testosterone**	1955	413	(21·1%)
**BMI**	1850	24·0	(21·5–27·5)
**Serum testosterone concentration at initiation of GAHT, nmol/L**^b^	1027	1·3	(1–1·7)
**Serum testosterone concentration during GAHT, nmol/L**^b^	1735	20·8	(13–32)
**Serum oestradiol concentration at initiation of GAHT, pmol/L**^b^	1037	168	(52·5–394)
**Serum oestradiol concentration during GAHT, pmol/L**^b^	1702	110·9	(71–166·0)
**Type of testosterone**			
Testosterone esters injections	1687	874	(51·8%)
Testosterone undecanoate injections		299	(17·7%)
Testosterone gel		430	(25·5%)
Other^c^		84	(5·0%)
**Other hormonal therapy (known use)**			
Progesterone	*-*	50	(2·6%)
Progesterone/estrogen		8	(0·4%)
GnRH-agonist		29	(1·5%)
**(former) smokers**	1288	776	(60·3%)

BMI during treatment: 94·6% of data available. Serum testosterone at initiation of GAHT: 52·5% of data available. Serum testosterone during treatment: 88·7% of data available. Serum oestradiol at initiation of GAHT: 53·0% of data available. Serum oestradiol during treatment: 87·1% of data available. Smoking: 65·9% of data available.

a Excluding missing.

b GAHT, gender-affirming hormone therapy.

c Testosterone undecanoate capsules.

Organ-specific characteristics are displayed in Table 2. Median duration of testosterone usage was 1·7 years (IQR 1·4–2·4) before hysterectomy and oophorectomy, and 3·1 (IQR 2·3–5·4) before vaginal and/or vulvar surgery or biopsy. Median age at time of surgery or biopsy was 24 years (IQR 20–33) for uterine and ovarian histopathology and 29 (IQR 22–39) for vaginal and vulvar histopathology. Uterine and ovarian histopathology had a median follow-up time (which equals testosterone exposure time) of 1·8 years (IQR 1·4–2·7), and vaginal and vulvar histopathology 5·1 years (2·4–14·5). In 42 uterine specimen (4·25%), testosterone exposure time equalled five years or more. Median age at time of histopathology acquisition was 32 years (IQR 24–37).

**Table 2:** Organ specific characteristics.

	Sample (N, total)^a^	Median (IQR) unless stated otherwise	
**Uterus**			
Time T—pathology (years)	989	1·7	(1·37–2·38) Min 1 Max 16·9
Age at time of pathology	990	24	(20–33)
Follow-up (years)	1853	1·8	(1·4–2·7) Min 1 Max 28·0
Total follow-up (person years)	1853	4742	
**Ovaries**			
Time T—pathology (years)	956	1·7	(1·37–2·38) Min 1 Max 24·39
Age at time of pathology	956	24	(20–33)
Follow-up (years)	1858	1·8	(1·4–2·7) Min 1 Max 28·0
Total follow-up (person years)	1858	4802	
**Vagina & vulva**			
Time T—pathology (years)	278	3·1	(2·3–5·4) Min 1 Max 39·4
Age at time of pathology	278	29	(22–39)
Follow-up (years)	1955	5·1	(2·44–14·5) Min 1 Max 34·2
Total follow-up (person years)	1955	18,380	

a Excluding missing.

Total specimens were 989 uteri, 1898 ovaries (from 956 individuals), 260 vaginal tissue resected at colpectomy, seven vaginal biopsies and 22 vulvar biopsies (see Fig. 1). No gynaecological malignancies were detected, precluding SIR calculation. Expected incidence was 0·26 or less for all cancer types (see Supplementary File S1). One ovarian serous borderline tumour, one case of simple endometrial hyperplasia and one case of lichen sclerosus with transformation to vulvar intraepithelial neoplasia III (VIN3) were detected. Based on the expected number of ≥VIN2 cases in our cohort (4·4) the age-adjusted standardised incidence ratio for > VIN2 was 0·23 (95% CI: 0·01–1·12). In three vulvar biopsies, lichen sclerosus was diagnosed without signs of malignant transformation. Based on an incidence rate of 14·6 per 100,000 person-years in the general population assigned female at birth,^28^ the standardised incidence ratio (not adjusted for age) for lichen sclerosis was 1·8 (0·7–4·1).

Of the pathology reports from uterine specimens, 545 included description of the endometrium. Sixty eight percent were described as inactive or atrophic and 32% as active (e.g. proliferative or secretory). In 543 cases, pathology reports of oophorectomy specimen included a detailed description of ovarian histology. Forty one percent included one or more recent signs of ovulation and/or ovarian activity. No correlation could be found between signs of ovarian activity and endometrial activity (Supplementary File S1). Table 3 shows the odds ratios of both univariate logistic regression per variables and the multivariate regression model. After adjustment for factors including age at time of surgery or biopsy and testosterone duration, both use of transdermal testosterone gel and intramuscular Testosterone esters were associated with active endometrium significantly more frequently compared to Testosterone undecanoate (OR 3·71; p = 0·02; 95% CI: 1·33–10·34 and OR 3·78; p = 0·01; 95% CI: 1·62–11·79, respectively).

**Table 3:** Univariate and multiple logistic regression analyses regarding potential factors associated with endometrial activity.

Variable	Univariate regression^a^				Multivariable regression^j^			
	Odds Ratio	p-value	95% CI		Odds Ratio	p-value	95% CI	
**Type of testosterone**^b^*RC: Nebido*								
**Gel**	1·99	0·08	0·92	3·66	3·71	0·02	1·33	10·34
**Sustanon**®^c^	1·83	0·08	0·91	4·35	3·78	0·01	1·62	11·79
**Age**^d^	0·98	0·12	0·96	1·0	0·96	0·05	0·92	1·00
**Duration T (years)**^e^	1·05	0·50	0·91	1·22	1·04	0·68	0·86	1·27
**BMI >30**^f^								
RC: BMI <29·9	0·91	0·76	0·50	1·66	0·81	0·64	0·34	1·91
**Ovarian activity**^g^	1·35	0·24	0·82	2·20	1·53	0·14	0·87	2·68
**Start GAHT with puberty blockers**^h^	0·90	0·74	0·47	1·70	0·48	0·11	0·19	1·18
**Before/after 2017**^i^	1·87	0·03	1·07	3·27	1·86	0·10	0·88	3·93

a After exclusion criteria, and without missing data, 420 cases were included in the model. Exclusion criteria: (other hormonal treatments (i.e. progesterone, combined oestrogen/progesterone or GnRH-agonists) and testosterone tablets).

b Reference category: Nebido® (Testosterone undecanoate injections); 380/420 (90%) of cases in model.

c Testosterone esters injections.

d Age at time of surgery or biopsy; 420/420 (100%) of cases in model.

e 420/420 (100%) of cases in model.

f Mean BMI during GAHT; Reference category: BMI <29·9; 401/420 (95%) cases in model.

g Signs of ovulation; 298/420 (71%) in model.

h 420/420 (100%) of cases in model.

i Reference category: after 2017; 420/420 (100%) of cases in model.

j 258/420 (61%) cases in model.

## Discussion

The aim of this retrospective cohort study was to study the effect of testosterone on the reproductive organs in TMGD individuals and to compare the incidence of gynaecologic (pre)-malignancies with the general population assigned female at birth.

No cases of endometrial, ovarian, vulvar or vaginal cancer were observed as was expected based on the age-adjusted incidence rates in the general population assigned female at birth. We found no increased risk of vulvar intraepithelial neoplasia ≥ VIN2 compared to the general population assigned female at birth.

The effects of testosterone on the endometrium of TMGD individuals remain poorly understood, with existing studies presenting varying results. Some studies report inactive endometrium in up to 100% of cases,^29^ while others document active endometrium in as many as 43–100% of patients.^30^ Our results support previous reports that testosterone does not induce atrophy in a substantial proportion of patients.

To improve our understanding of histopathological changes of the endometrium we investigated various factors potentially related to endometrial proliferation. No correlation was found between a high BMI and endometrial proliferation, contradicting earlier findings by da Silva et al., who reported an increase of endometrial activity in individuals with an elevated BMI.^31^ We did find a statistically significant association with endometrial activity for transdermal gel testosterone and intramuscular Testosterone esters compared to injectable Testosterone undecanoate. Though this may be spurious, it is biologically plausible that the variation in peak testosterone levels across different testosterone preparations may result in different levels of ovarian function suppression, and thus unopposed oestrogen exposure in the endometrium. Here, the elevated peak testosterone levels following Testosterone undecanoate injections may be more effective in suppressing ovarian activity. In contrast, shorter-acting intramuscular Testosterone esters are associated with greater fluctuations in serum testosterone levels, which may cause excess aromatisation, and permit intermittent ovarian activity during phases in which serum levels are lower.^32^ No association was found between ovarian activity and endometrial proliferation; however, only 543 out of 998 cases included comments on ovarian activity. Another potential factor regarding gel testosterone could be treatment adherence, as missed doses are more likely with daily dosed transdermal gel compared to the dosing schedule of 12 weeks of Testosterone undecanoate injections, resulting in less effective ovarian function suppression. Thus, endometrial activity may delineate those whose endometrial tissues have ongoing oestrogen exposure, the unopposed action of which is a risk factor for endometrial hyperplasia and cancer. Unfortunately, we lack temporal testosterone and oestradiol levels for this cohort, which would give an indication of medication concordance and true oestrogen exposure.

As discussed above, several causal pathways have been hypothesised for how testosterone may induce unopposed proliferation and increase risk of endometrial cancer. These include residual ovarian activity, aromatisation of testosterone to oestradiol and direct effect of testosterone on endometrial androgen receptors.^11^ Our understanding is complicated by the dynamic nature of both sex-steroid hormones and their receptors through the ovulatory cycle, and menopause, as well as differing aetiologies and hormone responsiveness of endometrial cancer subtypes.^33^

Endometrioid cancer, the most common subtype with 87% of cases,^34^ typically is oestrogen receptor positive, and lower grade, while other subtypes (serous, carcinosarcoma, clear cell and mixed type) express oestrogen receptors less commonly and are generally more aggressive.^35^

Multiple lines of evidence link testosterone to endometrial cancer development via the conversion to oestradiol. Higher levels of testosterone measured at single timepoint in premenopausal cisgender women have been associated with higher risk of endometrial cancer development post-menopause.^36^ However, this may not be reflective of lifetime exposure. Using Mendelian randomisation for variants in aromatase, higher predicted free testosterone levels were associated with adverse effects on both endometrioid and non-endometrioid endometrial cancer risk in cisgender women.^37^^,^^38^ However, it is worth noting that the levels of predicted testosterone in these studies are far lower than would be seen in TMGD individuals.

Regarding the direct effect of exogenous testosterone, this has been shown to reduce endometrial proliferation in cisgender women, regardless of menopausal status.^8^ The mechanism for this may be via displacement of oestrogen-regulated transcription factors by the androgen receptor, reducing oestrogen-driven cell cycle activation and thus inhibit endometrial cancer growth.^39^ However, in some contexts androgens may have a protumourigenic role, as seen in oestrogen receptor negative breast cancers.^40^ These findings fit with the observation that androgen receptors are highest in low-grade endometrioid tumour grade (with better prognosis) and are lowest in non-endometrioid histologic subtypes.^41^ The far higher levels of exogenous testosterone seen in TMGD individuals compared to cisgender women may therefore far outweigh any proliferative effects of aromatisation. This could explain why the majority of participants in our study had an atrophic endometrium.

Reassuringly, studies in the US utilising cancer databases have not shown an increased proportion of endometrial cancers in transgender patients, though this could be masked by the inability to verify for sex assigned at birth.^16^^,^^42^

However, if testosterone or its aromatisation of oestrogen does have protumourigenic effects on the endometrium, the length of testosterone exposure before hysterectomy is too short in most studies to-date, including our study, to be clinically informative.^43^ Future prospective studies are required to fully elucidate the long-term impact of high exogenous testosterone levels on endometrial cancer risk in TMGD individuals, considering the interplay of androgen receptors, histological subtypes, and hormone pathways.

No cases of ovarian malignancies were observed in this study, as was anticipated given the young age at time of ovariectomy in this cohort. One case of ovarian serous borderline tumour was found, occurring in an individual aged 39 years. Borderline tumours have low malignant potential and can be precursors of ovarian tumour across multiple histologies. These typically affect younger patients,^44^ as was the case in our study. Case reports of both ovarian carcinomas and borderline tumours in TMGD individuals, both with and without GAHT, are featured in the literature, often accompanied by speculation about the role of androgens.^18^^,^^20^ Androgen receptors are commonly expressed in high grade serous ovary cancer (the most common subtype, representing 75% of cases).^44^^,^^45^ However, androgenic signalling has been linked only to increased proliferation and not to tumourigenesis.^45^

Risk for epithelial ovarian cancer is increased by the number of ovulatory cycles rather than by hormone levels, and so risk may be lowered in TMGD individuals whose testosterone levels are adequate to suppress ovulation.^44^ In our study, however, we found almost half of patients having stigmata of ovulation.

Other studies have documented mainly morphological changes resembling those found in polycystic ovaries (PCO).^6^^,^^10^ These reports often used histopathologic criteria to diagnose PCO-like ovaries (e.g. collagenization of the outer cortex, stromal hyperplasia, luteinization of stroma cells and >12 antral follicles per ovary). However, inconsistencies in pathology reports frequently led to the omission of these criteria, and as a result, the number of PCO-like ovaries in this cohort was not documented. Additionally, significant inter-observer variability and the complex relationship between elevated testosterone levels and PCO complicate the interpretation of these morphological findings and their clinical relevance in this population.^6^^,^^12^^,^^17^

Given the suspected role of tubal pathology in the origin of high-grade serous ovarian carcinomas, opportunistic salpingectomy at the time of total laparoscopic hysterectomy (TLH) or sterilisation could be considered to reduce the risk of ovarian cancer within this population, aligning with broader recommendations for ovarian cancer prevention in individuals undergoing gynaecological surgery.^46^

Data on vaginal and vulvar pathology in TMGD individuals is scarce, with the exception of two case reports.^22^^,^^23^ The absence of malignancies and low number of ≥VIN2 in this cohort is reassuring. However, while no increased risk of vulvar or vaginal malignancies has been observed in the short term, there is biological plausibility that testosterone-exposed epithelium may be more susceptible to persistent high-risk HPV (hrHPV) infections, a known precursor to vaginal and vulvar cancers.^47^ Testosterone use has been linked to thinning of the vaginal epithelium and alterations in the vaginal microbiome, potentially compromising the epithelial barrier function and increasing the risk of chronic inflammation.^47^ Therefore, lowering barriers for HPV screening and improving vaccination rates amongst transgender individuals is paramount.^48^

The number of cases of lichen sclerosus (LS) in this cohort was relatively high compared to the incidence rates in cisgender women, although this did not reach statistical significance.^28^ LS is a chronic inflammatory skin-condition that primarily affects the vulvar and perineal areas. It can cause changes of the skin, such as atrophy, hyperkeratosis and sclerosis and is associated with an increased risk of vulvar squamous cell carcinoma.^49^ LS can cause vulvar pain and pruritus, symptoms also often associated with vaginal or vulvar atrophy, increasing the risk of misdiagnosis.^49^ Transmasculine individuals may avoid gynaecological examinations, potentially delaying diagnosis further. Given the malignant potential of LS, however, early recognition and treatment are crucial, emphasizing the need for clinical awareness.

A key strength of this study is its large cohort size of TMGD individuals and linkage to a pathology database with nationwide coverage to prevent incomplete follow-up. It is also the first study to investigate factors influencing endometrial activity in TMGD individuals using testosterone, which has shown wide variation in previous retrospective pathology studies.^6^^,^^10^, ^11^, ^12^^,^^29^

However some limitations should be taken into account, including significant missing data regarding well-recognised risk factors and mitigations for these cancer types, including hereditary cancer predisposition (ovary and endometrium), progesterone use (protective for endometrial cancer), and HPV status (risk factor for vulval and vaginal cancers).

The most significant limitation of this study is the young average age of this cohort, which may contribute to selection bias in this cohort. The median age of diagnosis for endometrial, epithelial ovarian (the most common type) and vulvar/vaginal cancers are 62, 63 and 70 respectively.^44^^,^^50^ This makes it challenging to determine differences in gynaecological cancer risk conferred by either long-term testosterone use or other factors e.g. screening behaviours, obesity etc. Another limitation is the reliance on textual pathology reports, without direct review of histological slides, which means that any inconsistencies of the pathological assessments cannot be ruled out completely. Lastly, our study is based on a Dutch cohort, which may not be fully representative of other populations, and the findings may not be entirely generalisable globally.

Further long-term co-produced prospective studies are required to study cancer risk and screening programmes in those electing to keep their gynaecological organs, while gynaecological evaluation in those experiencing abnormal uterine bleeding remains crucial. Inclusion of gender identity and trans status in cancer registries and secondary care records would also support a better understanding of the health of the TGD population. This also requires community involvement to ensure appropriate data security. Meanwhile, it is crucial not to over-speculate in the literature about risks of testosterone therapy in the absence of firm evidence as the potential harms of this include refusals of healthcare professionals to prescribe, over-screening of TMGD individuals, and misinformation that can bias individuals’ decision making around removal of these organs as part of gender-affirming surgery. The World Professional Association of Transgender Health Standards of Care 8 have recommended against removal of these organs for reduction in cancer risk alone.^51^

This study represents the largest cohort to date examining gynaecological histopathological findings in TMGD individuals. No cases of gynaecological malignancies were detected, although these findings align with expectations given the young age of the participants. We conclude that at a young age and with several years of testosterone use, the risk of gynaecological malignancies in TMGD individuals does not appear to be increased compared to the general population assigned female at birth. However, to better understand the long-term effects of testosterone on gynaecological organs and to provide informed counselling, further studies with extended follow-up and larger numbers of persons at typical cancer onset age are essential.

## Contributors

AV, WLJvV, CMW, and NMvM contributed to the study design and data collection. AV, WLJvV, AMB, CMW, and NMvM were involved in data analysis. AV, WLJvV, and AMB drafted the original manuscript. MLHS, MdH, FAG, JAFH, CMW, and NMvM reviewed and edited the manuscript. AV, WLJvV, NMvM, and CMW had access to and verified the underlying study data. All authors critically revised the manuscript, contributed to the data interpretation, and had final responsibility for the decision to submit for publication. AV and WLJvV share first authorship and CMW and NvM share last authorship.

## Data sharing statement

Statistics Netherlands prohibit data sharing at an individual level to guarantee the anonymity of the people in its databases.

## Declaration of interests

All authors hereby attest that they do not have any relevant conflict of interest related to this article.
