# Supplementary material for: Incidence of gynaecological (pre-)malignancies and endometrial activity in transmasculine and gender diverse individuals using testosterone: a retrospective, single-centre cohort study
Source: eClinicalMedicine. 2025 May 12;84:103248. doi: 10.1016/j.eclinm.2025.103248 (PMC12273736; doi:10.1016/j.eclinm.2025.103248)
Supplement: Supplementary Tables [file mmc1.pdf]

Supplementary Materials

SF Table 1 . Expected number of malignancies per type of cancer.

Type of cancer	Expected number	95% CI lower bound*	95% CI upper bound
**Endometrial cancer**	0·17	0	0·96
**Vulvar cancer**	0·26	0	1·27
**Ovarian cancer**	0·11	0	0·76
**Vaginal cancer**	0·05	0	0·46

* negative values were truncated given the nature of the data

SF Table 2 Characteristics Active Vs. Inactive endometrium

		**Active endometrium (n=175)**	**Inactive endometrium (n=370)**	**P-value**
**Age at time of pathology 0 missing**		25 (21-31)	24 (21-34)	0·77 [α]
**Time T – pathology (years) 0 missing**		1·7 (1·4–2·6)<br>Min 1<br>Max 11·6	1·8 (1·4-2·5)<br>min 1<br>max 12·2	0·95 [α]
**Puberty-blockers before start T**		23 (13·1%)	68 (18·4%)	0·13[β]
**Type of T[1]**	**Testosterone undecanoate injections**	13 (8·2%)	52 (15·1%)	**0·04** [β]
	**Testosterone esters injections**	92 (57·9%)	210 (60·9%)	
	**Testosterone gel**	39 (24·5%)	61 (17·7%)	
	**Other ***	15 (9·4%)	22 (6·4%)	
**Other hormonal therapy**	**Progesterone**	21	30	0·91 [β]
	**Oestrogen & Progesteron**	2	1	
	**GnRH-agonist**	9	34	
**Mean BMI during GAHT[3]**		23·7 (IQR 21·8–27·1)	24·3 (IQR 22·0–27·2)	0·37 [α]
**Serum testosterone concentration <365 days to PA, nmol/L[6]**		22 (IQR 12·0–37·0)	22·5 (14·0–34·0)	0·99 [α]
**T groups**	**<10**	14 (18·4%)	28 (14·1%)	0·25 [β]
	**10-30**	32 (42·1%)	106 (53·3%)	
	**>30**	30 (39·5%)	65 (32·7%)	
**Serum oestradiol concentration <365 days to PA, pmol/L[7]**		116·2 (IQR 64·0-201)	110·0 (IQR 63·0–175·4)	0·85 [α]
**E tertiles**	20-88	31 (41·9%)	69 (35·9%)	**0·05** [β]
	89-151	12 (16·2%)	60 (31·3%)	
	151·8-1490	31 (41·9%)	63 (32·8%)	
**Signs of ovarian activity[8]**		61 (49·6%)	112 (42·3%)	0·17 [β]

α Mann-Whitney-U test

β Chi-square test

* Testosterone undecanoate capsules

1. Active 91% data available; inactive 93% data available;
2. Active 66% data available; inactive 72% data available
3. Active 95% data available; inactive 95% data available
4. Active 49% data available; inactive 59% data available
5. Active 49% data available; inactive 57% data available
6. Active 43% data available; inactive 54% data available
7. Active 42% data available; inactive 52% data available
8. Active 79% data available; inactive 72% data available
